# Increased Urinary CD163 Levels in Systemic Vasculitis with Renal Involvement

**DOI:** 10.1155/2021/6637235

**Published:** 2021-04-30

**Authors:** Xiayire Aierken, Qing Zhu, Ting Wu, Sha sha Liu, Yuan yuan Cao, Xin tian Cai, Ayiguzaili Aihemaiti, Yunwei Bi, Shunfan Yang, Weiwei Zhang, Nanfang Li

**Affiliations:** Hypertension Center of People's Hospital of Xinjiang Uygur Autonomous Region, Xinjiang Hypertension Institute, National Health Committee Key Laboratory of Hypertension Clinical Research, Urumqi, Xinjiang 830001, China

## Abstract

**Objectives:**

Systemic vasculitis includes a group of disorders characterized by inflammation of the vessel wall, involving multiple systems, and can cause malignant hypertension. CD163 is a specific marker of anti-inflammatory macrophages. This study is aimed at evaluating the CD163 levels in relation to systemic vasculitis and renal involvements.

**Methods:**

Urinary CD163 levels were retrospectively measured by enzyme-linked immunosorbent assay (ELISA) in 51 patients with systemic vasculitis, 42 essential hypertensions, and 36 healthy volunteers. The associations between urinary CD163 levels and clinical indicators were analyzed.

**Results:**

Urinary CD163 levels were significantly higher in patients with systemic vasculitis [68.20 (38.25~158.78) (pg/ml)] compared to essential hypertension [43.86 (23.30-60.71) (pg/ml)] (*p* = 0.003) and the healthy volunteers [30.76 (9.30-54.16) (pg/ml)] (*p* < 0.001). Furthermore, systemic vasculitis patients with renal involvement had significantly higher urinary CD163 levels relative to patients without renal involvement [86.95 (47.61 and 192.38) pg/ml] vs. [41.99 (17.70 and 71.95) pg/ml, *p* = 0.005]. After control factors age, sex, and BMI, urinary CD163 levels in systemic vasculitis patients were positively correlated with serum creatinine, blood urea nitrogen, and *β*-2 microglobulin (*r* = 0.45, 0.48, and 0.46; *p* = 0.001, 0.001, and 0.002, respectively). In addition, we found the level of urinary CD163 in granulomatous vasculitis (including TA, GPA, and EGPA) was significantly higher than that in necrotizing vasculitis (including PAN) [86.95 (41.99 and 184.82) pg/ml] vs. [45.73 (21.43 and 74.43) pg/ml, *p* = 0.016].

**Conclusion:**

Urinary CD163 levels were significantly higher in patients with systemic vasculitis, especially in patients with renal involvement. Thus, urinary CD163 has the potential to be a biomarker for systemic vasculitis with renal involvement.

## 1. Introduction

Vasculitis includes a group of disorders characterized by inflammation of the vessel wall, and it is classified based on the diameter of the predominantly involved vessels, etiology, and extent of involvement. The pathogenesis of vasculitis is related to inflammatory cells, and these cells infiltrate the vessel wall, phagocytose the immune complexes, release their intracytoplasmic enzymes, and will eventually lead to lesion of the vessel lumen with ischemic changes [1, 2]. Vasculitis can involve a variety of systems, leading to differences in its clinical manifestations. In some cases, hypertension is one of the main clinical manifestations of vasculitis. The study has shown that vasculitis is one of the most important causes of malignant hypertension [3]. If untreated, this group of diseases can pose a threat to patient's life. However, due to the heterogeneity of clinical manifestations, unclearness of the pathogenesis and the traditional biomarker like CRP (C-reactive protein), ESR (erythrocyte sedimentation rate), interleukin family, or imaging examinations failed to diagnose and evaluate the vasculitis timely [4]. Meanwhile, the prognosis remains difficult to predict. It often comes to the attention of clinicians after the decline of organ function. Therefore, laboratory markers that can help clarify the pathogenesis and monitor the systemic vasculitis are urgently needed.

CD163, also known as the hemoglobin (Hb) scavenger receptor, is a macrophage-specific protein, composed of nine cysteine-rich domains [5]. Upregulated expression of this receptor is one of the major changes in the macrophage switch to alternative activated phenotypes in inflammation. Accordingly, a high CD163 expression in macrophages is a characteristic of tissues responding to inflammation [6]. Furthermore, there were a certain number of studies that investigated the links between CD163 with the occurrence and development of autoimmune diseases. Previous researches have shown that CD163 significantly increased in rheumatoid arthritis, lupus nephritis, systemic sclerosis, etc., indicating it may play a significant role in the pathogenesis of immune disorder and inflammation [7–9]. Urine CD163, as a noninvasive biomarker, has been found to be associated with renal involvement in autoimmune diseases and showed higher sensitivity and specificity than the serum CD163 levels [10]. Renal involvement is a common clinical manifestation of ANCA-associated vasculitis [11]. Therefore, the previous studies of urinary CD163 levels in vasculitis mainly focused on AAV. However, to our knowledge, urinary CD163 levels in patients with other subtypes of vasculitis have not been studied. Therefore, we aim to investigate the concentration of urinary CD163 in patients with different types of vasculitis and relate this measurement to clinical parameters.

## 2. Methods

### 2.1. Participants

This cross-sectional study was included three groups of people. All participants were recruited from People's Hospital of Xinjiang Uygur Autonomous Region between January 1, 2017, and December 31, 2019.

*Systemic vasculitis (SV)*: the cases of 51 patients with systemic vasculitis, including 20 Takayasu arteritis (TA), 13 polyarteritis nodosa (PAN), and 18 ANCA-associated vasculitis (AAV), were recruited. Patients selected from our center who underwent vessel biopsy or angiography, after combining with clinical manifestations and laboratory examination, fulfilled the 1990 American College of Rheumatology (ACR) criteria for TA, PAN, or the 2012 revised International Chapel Hill Consensus Conference classification criteria for ANCA [12–14]. Patients who had secondary vasculitis, systemic lupus erythematosus, rheumatoid arthritis, malignancy, any form of acute or chronic infection, or any other coexisting renal disease, such as antibasement membrane glomerulonephritis, IgA nephropathy, diabetic nephropathy, or lupus nephritis, were excluded.

*Essential hypertension (EH)*: this group of participants diagnosed with hypertension, according to diagnostic criteria of 2018 revised Chinese guidelines for the Prevention and Treatment of Hypertension [15]. (Hypertension was defined as follows: without using antihypertensive drugs, SBP ≥ 140 mmHg and/or DBP ≥ 90 mmHg were measured three times in different days. The patient has a history of hypertension and is currently taking antihypertensive drugs, although the blood pressure is lower than 140/90 mmHg, still be diagnosed as hypertension.) Then, these patients completed a series of screening for secondary hypertension, such as renal hypertension, renovascular hypertension, primary aldosteronism, pheochromocytoma, and severe sleep apnea syndrome, and eventually, 42 patients diagnose with essential hypertension were selected. Patient with rheumatic diseases, diabetes, malignant tumors, history of infection within one month, and kidney diseases was not included.

*Healthy volunteer (HV)*: 36 healthy volunteers with the auxiliary examination are basically negative (including blood routine, urine routine, liver, renal function, inflammatory index, and chest X-ray) and were recruited from the Center for Medical Examination of our hospital. Those who have history of hypertension, renal diseases, rheumatic diseases, diabetes, and hyperlipidemia were excluded. Meanwhile, through questionnaires and laboratory indicators, we exclude infections of respiratory, gastrointestinal, and urinary tract within one month.

### 2.2. Ethics Approval of the Study Protocol

Written informed consent was obtained from all participants who provided permission for collection of relevant clinical data and urine sample. This study was approved by the Ethics Committee of the People's Hospital of Xinjiang Uygur Autonomous Region (Urumqi, China) and was conducted according to the standards of the Declaration of Helsinki. The approval number is KY2018011866.

### 2.3. Collection of Urine Sample and Measurement of Urinary Levels of CD163

Urine samples were collected before any form of invasive renal examination including renal biopsy or renal angiography. Seven (7/51) patients were treated with glucocorticoids, and two of them were treated with immunosuppressants (hydroxychloroquine phosphate 0.2 g qd and cyclophosphamide 0.4 g wd) before sample collection. The middle of the morning urine was collected in sterile cups after 8 hours on an empty stomach and centrifuged for 15 minutes at a rotational speed of 3000 rpm at room temperature, and the supernatant was taken and subpacked in three 5 ml tubes and stored at -80°C for detection.

Samples were placed at -20° refrigerator for one night before the test, and the 37° incubator was used before assay procedure. The protein expression level of CD163 in urine was detected by commercially available enzyme-linked immunosorbent assay (ELISA) (Duoset DY1607; R&D Systems, Minneapolis, MN, USA) according to the manufacturer's protocol. Absorbance was measured in a microtiter plate reader (Bio-Rad, Hercules, CA, USA) at a wavelength of 450 nm. Duplicate readings were averaged for each standard, control, and sample, and the average zero standard optical density was subtracted. Best-fit lines were drawn through the standard points using the plotting software. When out of the measuring range, sample was diluted at 1 : 4, and the concentration read from the standard curve has been multiplied by the dilution factor.

### 2.4. Data Collection

All clinical data including demographic, clinical, biologic, imaging, and biopsy findings were obtained from the patient's medical records during hospitalization. Biologic parameters that were recorded included blood routine, urine routine, blood coagulation function, renal parameters [proteinuria, hematuria, 24 h proteinuria, urinary microalbuminuria, N-acetyl-beta-D-glucosaminidase (NAG), blood urea nitrogen (BUN), and serum creatinine (Scr)], transaminase level, erythrocyte sedimentation rate (ESR), levels hypersensitive CRP (Hs-CRP), serum homocysteine, glycosylated hemoglobin, and thyroid hormone level. In imaging examinations, angiography results were classified as abnormal when blood vessels were sparse and/or irregular stenoses and/or microaneurysms were present. Chest X-rays that showed nodules, infiltrating lesions, and/or cavity were also classified as abnormal. The results were independently determined by 2 radiologists. Vasculitis was confirmed in biopsy findings for patients who showed inflammatory cell infiltration in small, medium vessel, and/or formation of crescents. Immunofluorescence analysis of biopsy samples confirmed vasculitis when no or little immune complex deposition was present in the mesangial area, vascular loops, or small vascular walls. The results were independently determined by 2 pathologists.

### 2.5. Definition of Renal Involvement

Vasculitis with renal involvement was determined by the physician-investigator and was informed by showing any one of (1) urine protein levels > 0.2 g/L, (2) hematuria of 1+ (>10 at high magnification) excepting menstrual period interference, (3) 24 h proteinuria > 0.141 g/L, and (4) Scr > 104 and 84 *μ*mol/L for male and female, respectively [16].

### 2.6. Statistical Method

Analyses were performed using the SPSS software version 23.0 (IBM Corp., Armonk, NY), and graphs were built using Graph Pad Prism version 5.0 (GraphPad Software, La Jolla, CA). Data are expressed as mean ± standard deviation for data that were normally distributed (like age, blood pressure, BMI, blood routine, and uric acid), median and interquartile range for data that were not normally distributed (like ESR, Hs-CRP, Scr, 24 h proteinuria, urinary microalbuminuria, and urine CD163) or as a percentage for categorical variables. Differences in quantitative parameters between groups were assessed using ANOVA test and *t*-test (for data that are normally distributed), nonparametric tests, Kruskal–Wallis and Mann–Whitney *U* tests (for data that are not normally distributed), or *χ*^2^ test (for categorical variables). A correlation analysis was performed using Spearman's or rank correlation coefficient (*r*) to determine correlations between the urine levels of CD163 and clinical parameters. Logistic regression was used for assessment of confounding factors. Receiver operating characteristic (ROC) curve analysis was used to identify diagnostic values for combination of CD163 and other clinical parameters. *p* values <0.05 were considered to be statistically significant.

## 3. Results

### 3.1. Baseline Characteristics of Patients with Vasculitis and Control Groups

The demographic characteristics and laboratory features of 51 systemic vasculitis patients, 42 EHs, and 36 HVs were shown (Table 1). The median age of the systemic vasculitis patients was 42.56 years (range 19-81 years), and among them, male/female ratio was 1.94. When comparing the three groups, it was found that blood pressure level, BMI, white blood cell count, mononuclear cell count, ESR, and serum creatinine (Scr) levels were all significantly higher in vasculitis patients than in control groups. When compared to essential hypertension, we also found systemic vasculitis patient with elevated urinary microalbuminuria level and higher positive rate of proteinuria and hematuria (Table 1). Transaminase had no difference between the three groups. And there was no difference between serum homocysteine, glycosylated hemoglobin, thyroid hormone level, etc. between vasculitis and essential hypertension groups (data not shown).

### 3.2. Urinary CD163 Levels in Patients with Vasculitis

**Figure 1:**
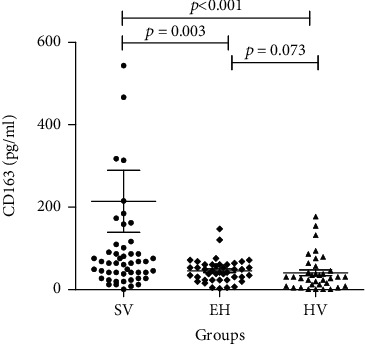
Urinary CD163 level in different groups. The levels of urinary CD163 were measured in 53 systemic vasculitis (SV), 42 essential hypertension (EH), and 36 healthy volunteers (HV). Data shown are the interquartile range of individual subjects. The data were analyzed by the Kruskal–Wallis test.

Urinary CD163 levels were significantly higher in patients with systemic vasculitis [median 68.20, IQ (38.25~158.78) (pg/ml)] compared to essential hypertension [median 43.86, IQ (23.30-60.71) (pg/ml)] (*p* = 0.003) and the healthy volunteers [median 30.76, IQ (9.30-54.16) (pg/ml)] (*p* < 0.001). There was also significant difference between EH and HV (*p* < 0.001) (Figure 1). When logistic regression was used to correct for gender, age, blood pressure, and BMI, the difference of CD163 among three groups was still significant (*p* = 0.036).

**Figure 2:**
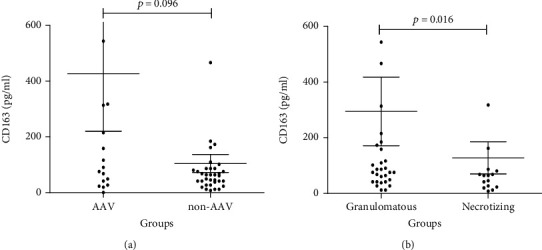
Urinary CD163 level in different vasculitis groups. Urinary CD163 in 18 AAV and 35 non-AAV. (a) Urinary CD163 in 31 granulomatous vasculitis and 13 necrotizing vasculitis. (b) Data shown are the interquartile range of individual subjects. The data were analyzed by the Mann–Whitney *U* test.

When we divided the patients with systemic vasculitis into two groups [AAV group and non-AAV groups (including TA and PAN)] and compared the urinary CD163 levels of the two groups, we found that the difference was not statistically significant [AAV median 103.85, IQ (38.25-374.15) vs. non-AAV median 64.45, IQ (32.64-86.95), *p* = 0.096] (Figure 2(a)). In addition, according to the different pathological mechanisms, we divided the patients with systemic vasculitis into two groups [granulomatous vasculitis group (including TA, GPA, and EGPA) and necrotizing vasculitis group (including PAN and MPA)] and the urine CD163 level of the two groups was analyzed again. Interestingly, the level of urinary CD163 in granulomatous vasculitis [median 86.95, IQ (41.99 and 184.82)] was significantly higher than that in necrotizing vasculitis [median 45.73, IQ (21.43 and 74.43)], *p* = 0.016 (Figure 2(b)).

### 3.3. Correlation of Urinary CD163 Levels with Clinical Parameters

**Figure 3:**
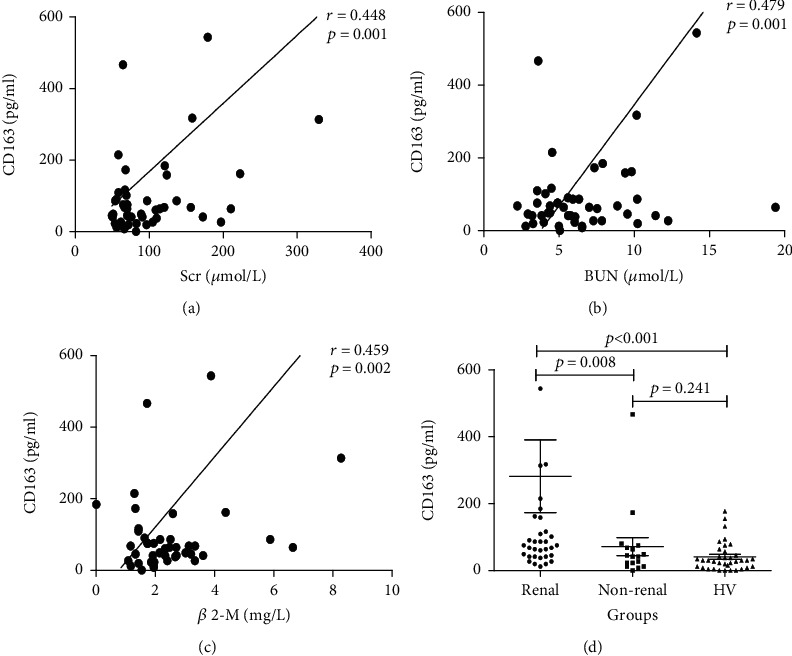
The correlations of urinary CD163 with (a) serum creatinine (Scr), (b) blood urea nitrogen (BUN), and (c) *β*-2 microglobulin (*β*-2-M) in systemic vasculitis patients. Urinary CD163 levels in systemic vasculitis patients with renal involvement and without renal involvement. (d) The potential association between them was analyzed by the Spearman rank correlation. Subgroup was analyzed by the Mann–Whitney *U* test.

We analyzed the correlation of urinary CD163 levels with clinical indicators. Notably, there were significant positive correlations of urinary CD163 levels with serum levels of urea nitrogen, Scr, and *β*-2 microglobulin. After control factors age, sex, and BMI, results were still statistically significant. However, there was no correlation between urinary CD163 and microalbuminuria or NAG. When further analysis is conducted according that whether the kidney is involved or not, CD163 levels higher in patients who had renal involvement [median 86.95, IQ (47.61 and 192.38)] than in those who did not [median 41.99, IQ (17.70 and 71.95)], the difference between the two groups is significant (*p* = 0.008). The nonrenal involvement group was higher than the HV, but there was no statistical difference (*p* = 0.241) (Figure 3).

#### 3.3.1. ROC Curve of Urine CD163 in the Diagnosis of Systemic Vasculitis

**Figure 4:**
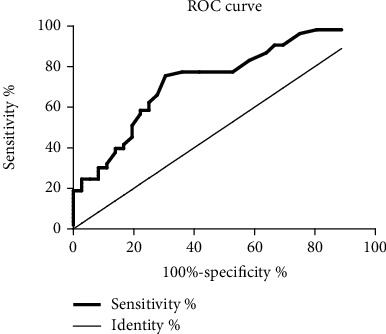
ROC curve of urinary CD163 in the diagnosis of systemic vasculitis.

The results showed that urine CD163 was effective in differentiating systemic vasculitis with an area under the ROC curve of 0.738. The cutoff value is 40.12 pg/ml, and sensitivity and specificity were 74% and 69.4%, respectively, as shown in Figure 4.

## 4. Discussion

Although with the improvement of detection technology and attention to autoimmune diseases, the diagnosis rate of vasculitis has improved, but it still remains a major challenge due to concealment of disease, low completion rate of biopsy, generality and nonspecificity of clinical symptoms, and lacking of specificity and sensitivity biomarkers. Thus, potential biomarkers are urgently needed in the diagnosis and monitoring of vasculitis [17]. Substantial evidence points toward monocytes and macrophages playing prominent roles in early stage of disease, mediating both pro- and anti-inflammatory responses [18]. In this study, we found mononuclear cell level was also higher expressed in SV patients. Taken together, it is suggested that mononuclear-macrophage system may be involved in the pathogenesis of vasculitis, leading to the disorder of proinflammatory and anti-inflammatory processes. Due to the high and specific expression on macrophages, CD163 has gained much attention as a potential biomarker reflecting macrophage activation in inflammatory conditions. CD163 as a marker of macrophages has innate advantages in deepening our understanding of vasculitis. van Sleen et al. studied 41 patients diagnosed with GCA and found the level of serum CD163 in the GCA was significantly increased and related to severity of disease [19]. Alpsoy et al. found that CD163 may be involved in the repair or anti-inflammatory process of vascular injury in Behçet's disease [20]. However, previous studies mainly focused on the level of CD163 in the blood. Recent research has found that urine as specimen source of biomarker has a unique advantage over blood. Not only can be completely noninvasive but also does not have a steady-state regulation mechanism. In the early stages of the disease, the blood is still under the control of the steady-state regulation mechanism, and early characteristics of the disease are not shown in the blood. But these early pathological changes can be retained in the urine [21]. In our study, we found that urinary CD163 concentrations were significantly elevated in patients with vasculitis when compared to healthy controls. This was consistent with the conclusion that previous studies have found that the expression of urinary CD163 is increased in vascular inflammation.

In addition to being secreted by monocytes, CD163 can also be secreted by other immune cells such as endothelial cells, and our patients with vasculitis often have high blood pressure. Therefore, in order to exclude the influence of the factors, we also compared and analyzed urinary CD163 levels in the vasculitis group and essential hypertension group. The results showed that the urinary CD163 levels in vasculitis group were higher than essential hypertension. And through regression analysis correcting age, blood pressure, and BMI, the difference is still significant. But there was no difference between essential hypertension and healthy volunteers. It suggested that there were differences in the cells and cytokines involved in the process of vascular injury caused by vasculitis and hypertension, and CD163 may be a relatively specific indicator of vasculitis.

CD163 has been shown to be upregulated in AAV. Previous research has identified several urinary biomarkers that may be useful for monitoring AAV, including the levels of monocyte chemoattractant protein-1 (MCP-1) and high mobility group box-1 (HMGB1). MCP-1 is produced by renal cells and monocyte stimulated by inflammation, which may cause renal fibrosis through macrophage recruitment and direct induction of glomerular mesangial cell fibrosis, which increases synchronously with CD163 in patients with AAV [22]. In the state of vascular inflammation, the complex of HMGB1 and hemoglobin binds to CD163, activating monocytes and promoting the expression of inflammatory factors [23]. Recently, the urinary concentration of activin A was found to be a biomarker for AAV and was also associated with the degree of CD163-positive macrophage infiltration [24]. In our result, patients with AAV have the trend of increasing CD163 levels, but not showing statistical difference between subgroups of vasculitis, and it may relate to the great disparity in the number of the two groups (18 vs. 35) and small sample size. Also, another possibility cannot be ignored. The link between CD163 and AAV has been found before, and it may relate to AAV that involves the kidneys more often. Therefore, it is necessary to further expand the sample size to testify if this rising trend makes sense.

When grouped according to different pathological mechanisms, we unexpectedly found that CD163 levels were significantly higher in granulomatous vasculitis than in necrotizing vasculitis. It suggested that CD163 was not a specific indicator of AAV, and CD163 may be also involved in the pathogenesis of other vasculitis, such as TA, BD, and GCA, but not PAN. The histopathologic feature of granulomatous vasculitis has provided evidence to support a role of pathogenic T lymphocyte responses and cell-mediated immune injury. Vascular endothelial cells activated by cytokines, such as interferon (IFN) *γ*, secrete cytokines like IL-1 and TNF-*α*, stimulate T lymphocytes, and initiate or propagate in situ immunologic processes within the blood vessel [1]. Macrophages play a critical role in the disease pathogenesis. This also shows that our results are reasonable.

As the one of the most commonly affected organs by systemic vasculitis, the accurate evaluation of the kidney function is very important in the monitoring of vasculitis patients. All parts of the kidney unit are involved in the production of urine. Therefore, urine is the most direct assessment tools on the function and state of the urinary system [21]. Previous studies have found that urinary CD163 may be associated with kidney damage. When distinguishing patients with active renal vasculitis, the AUC of urine CD163 was 0.9, with the sensitivity of 96% and specificity of 94% [9]. Latest research showed that in systemic lupus erythematosus, the concentration of urinary CD163 can increase hundreds of times when the kidney is involved [8]. In principle, in the healthy state without glomerular basement membrane damage, the concentration of CD163 in urine should be very low due to the high molecular weight (130 kDa). To explore the changes of urinary CD163 in patients with vasculitis who have renal involvement, we divided vasculitis into two groups: renal involvement and nonrenal involvement. The results showed difference in subgroups, and the level of CD163 was significantly increased in patients with renal involvement. When we analyzed the correlation of urinary CD163 levels with clinical parameters, we found that CD163 is correlated with the levels of kidney injury markers, like Scr, BUN, and *β*-2-M. This further confirmed our conclusion. Taken together, these results suggest that urinary CD163 concentration may be a useful biomarker for monitoring renal involvement in patients with SV.

Our study has several advantages. As we know, this is the first research to determine the concentration of urinary CD163 levels in various vasculitis. Secondly, it is also the first time that urinary CD163 has been compared between vasculitis and hypertension. Previous studies were mainly limited to ANCA-associated vasculitis and healthy control. Third, according to strict exclusion criteria, we exclude diseases that have a clear impact on CD163 levels in previous studies, in order to improve the accuracy of the experimental results, which also limits our sample size. And several limitations of the present study should be mentioned. First, the study was a retrospective design with small sample size. Secondly, AUC of CD163 was not enough high (0.738) as a biomarker in clinical practice. This result might be associated with low sample number and heterogeneity in background diseases. The sample size needs to be expanded in the further study to confirm the reliability of urine CD163 as a biomarker. Thirdly, there is no good consistency in demographic characteristics. Fourthly, it is because the expression of CD163 is upregulated by glucocorticoid the serum CD163 levels of some patients treated with glucocorticoids before initial sample collection, but it accounts for a very small part (7/51), and there is no difference between with or without glucocorticoid therapy (*p* = 0.398).

In conclusion, the present study showed usefulness of urinary CD163 as a biomarker for SV, especially those with impaired renal function. It can provide some reference value as a macrophage activity marker complementary to clinical findings and other laboratory tests. Certain positive results were obtained in this study. However, whether CD163 can be used as a biomarker needs to be determined in prospective and larger clinical samples, and the exact role of CD163 in the pathogenesis of SV needs to be clarified.

## Figures and Tables

**Table 1:** Demographic and laboratory features of patients with systemic vasculitis and control groups.

Variables	Systemic vasculitis	Essential hypertension	Healthy controls	*p* value
Demographic characteristics				
Age	41.46 ± 10.69	52.62 ± 11.85	26.36 ± 1.93	0.001
Male (*n*)	33	24	12	0.001
SBP (mmHg)	157.28 ± 25.03	146.19 ± 26.44	117.61 ± 12.05	0.001
DBP (mmHg)	100.96 ± 22.04	90.98 ± 18.24	70.03 ± 7.91	0.001
BMI (kg/m^2^)	25.08 ± 3.85	25.39 ± 2.97	21.85 ± 3.40	0.001
Laboratory test				
Hb (^∗^10^12^)	139.30 ± 21.72	141.79 ± 16.77	145.28 ± 18.53	0.311
WBC (^∗^10^9^)	7.59 ± 2.16	6.04 ± 1.55	6.39 ± 1.38	0.001
PLT (^∗^10^9^)	252.88 ± 70.47	228.07 ± 45.99	288.61 ± 55.87	0.688
Mono cell^∗^ (10^9/l^)	0.51 ± 0.17	0.43 ± 0.15	0.45 ± 0.14	0.018
ESR (mm/h)	20.00 (6.00, 32.00)	12.00 (8.50, 22.00)	12.00 (7.25, 16.00)	0.047
Hs-CRP (mg/L)	2.50 (0.89, 6.24)	1.34 (0.6, 2.54)	—	0.130
Scr (*μ*mol/L)	78.83 (65.76, 141.78)	61.00 (54.00, 70.15)	75.78 (66.60, 87.24)	0.001
Uric acid (*μ*mol/L)	388.52 ± 121.02	327.11 ± 98.54	—	0.007
Proteinuria (*n*)	21 (41.18%)	11 (26.2%)	0 (0%)	0.001
Hematuria (*n*)	4 (7.84%)	0 (0%)	0 (0%)	0.001
Microalbuminuria (mg/L)	24.50 (10.25, 97.40)	5.60 (5.00, 9.09)	—	0.001
24 h proteinuria (g/d)	2.80 (0.11, 5.01)	0.31 (0.14, 1.69)		0.374
NAG	3.13 (1.16, 8.18)	4.69 (3.09, 6.29)	—	0.160
Glucocorticoids (*n*, mg/d)	7 (10-40 mg)	—	—	—
Immunosuppressants (*n*)	2	—	—	—

Annotation: SBP: systolic blood pressure; DBP: diastolic blood pressure; BMI: body max index; RBC: red blood cell; WBC: white blood cell; PLT: blood platelet; ESR: erythrocyte sedimentation rate; Hs-CRP: hypersensitive C-reactive protein; Scr: serum creatinine; NAG: N-acetyl-beta-D-glucosaminidase.

## Data Availability

There is no use of public database. The original data of the experiment can be obtained by contacting the correspondence author by email.
